# Weathering words: a virtual reality study of environmental influence on reading dynamics

**DOI:** 10.3389/fpsyg.2024.1433781

**Published:** 2024-10-14

**Authors:** Francisco Rocabado, Laís Muntini, Jorge González Alonso, Jon Andoni Duñabeitia

**Affiliations:** ^1^Centro de Investigación Nebrija en Cognición (CINC), Department of Education, Universidad Nebrija, Madrid, Spain; ^2^Center for Cognitive Science, University of KaiserslauternLandau, Kaiserslautern, Germany; ^3^Center for Language, Brain and Learning (C-LaBL), UiT The Arctic University of Norway, Tromsø, Norway

**Keywords:** virtual reality, ecological validity, lexical decision task, reading disfluency, visual noise

## Abstract

**Introduction:**

Reading is a fundamental cognitive activity that is influenced by both textual and external environmental factors, although the latter has been less thoroughly explored. This study aims to examine the impact of environmental visual conditions on reading performance using Virtual Reality (VR) technology.

**Methods:**

We conducted two experiments to assess the effects of visual contrast and simulated weather conditions on reading dynamics. In Experiment 1, we measured single-word recognition speed using a lexical decision task under different visual contrasts and weather conditions. In Experiment 2, we assessed reading dynamics during a sentence reading task, analyzing how visual contrast and simulated sunny versus rainy weather conditions affected reading behavior, particularly focusing on reading speed and eye fixations.

**Results:**

In Experiment 1, high visual contrast, particularly under sunny conditions, significantly enhanced single-word recognition speed, indicating a notable influence of environmental visual conditions. In Experiment 2, visual contrast had minimal effect on sentence reading; however, sunny weather facilitated faster reading times, while rainy scenarios increased the number of eye fixations.

**Discussion:**

These findings suggest that environmental factors, such as weather conditions, can significantly affect reading behavior. The study contributes to the understanding of key environmental influences on reading in everyday life contexts and has implications for the ergonomic design of reading materials, especially for outdoor settings and VR environments. Additionally, the integration of controlled stimuli within VR increases the ecological validity of reading research, underscoring the potential of VR as a powerful tool for cognitive research.

## 1 Introduction

From a glance at a street sign to an immersive experience of a novel, reading is omnipresent. We are routinely exposed to a wide range of reading materials, ranging from display types on traffic signs and printed text in novels to newspaper headlines, handwritten notes, and ever more frequent, digital content on digital displays. Textual markers, bearing both informative and commercial purposes, guide our navigation in the world. However, how these materials are perceived in everyday contexts is not only determined by their design (e.g., font, materials, etc.), but is also influenced by environmental—meteorological and other—factors such the blur created by raindrops, the haze of fog or a sandstorm, or the accumulated dirt of dust. While the intrinsic properties (e.g., word frequency, orthography, or length) of reading stimuli have been widely studied in psycholinguistics, there is a knowledge gap regarding environmental variables that surround us and are present in our everyday lives. Here, we rely on the Virtual Reality (VR) technique, as it offers a reliable way to reproduce such environmental conditions through controlled, realistic, and immersive 3D scenarios, to explore the effect of weather conditions on single word and sentence reading.

In the context of cognitive research, the term fluency describes the subjective perception of ease or difficulty experienced while engaging in a mental task. This has a bearing on strategic decision-making, leading individuals to employ different cognitive approaches based on the perceived fluency of the information presented (Oppenheimer, 2008). Building upon this concept, various intrinsic and extrinsic factors have been identified as determinants of single-word reading fluency. These encompass lexical attributes such as word length and frequency (e.g., Aguasvivas et al., 2020), physical characteristics like font size and contrast (Bernard et al., 2003; Schindler et al., 2018), ambient luminance (Dobres et al., 2018), and surrounding distractors proximate to the focal target (Kemper et al., 2008). However, Dobres et al. (2018), point out that real-world interface effects are more pronounced than in-script effects (e.g., font size) when it comes to overall word processing. Reading changes in our surroundings could lead to increased reading costs (e.g., switching from reading road billboards to street signs), given that adapting to the mental representation from one reading format to another requires effort. This underscores the complexity of external factors present in real-life reading situations. However, studying such extralinguistic features consistent with real-life reading behaviors (e.g., traffic signs, advertisement billboards) are challenging to reproduce in traditional laboratory settings.

The manipulation of different word features and degradation of the linguistic input (visual noise) has helped researchers identify some of the variables that lead to reading disfluency, affecting language processing, and reading times. An important tool in this line of word recognition research is the Lexical Decision Task (LDT, Meyer and Schvaneveldt, 1971). This task requires readers to classify a briefly presented string of letters as either a word or a nonsense pseudoword. The most basic and robust of all effects associated with the LDT is the lexicality effect, whereby readers recognize words faster and more accurately than pseudowords. By varying the characteristics of the stimuli presented and analyzing differences in participant’s response accuracy and reaction times (RTs), reading research employing the LDT has contributed to the mapping of some of the main in-script factors modulating word recognition (Balota and Chumbley, 1984). A prominent factor that challenges the reader’s ability to recognize and process words is the introduction of visual noise, such as pixel disruptions or blurring (Gagl et al., 2020). By manipulating visual noise, reading researchers took a further step in understanding visual word recognition, by introducing a simulation of imperfect real-world reading conditions.

In parallel to single-word processing in tasks requiring a lexical decision, research on reading has also investigated the effects of script manipulation on legibility and its subsequent influence on eye movement patterns. For instance, Rayner et al. (2006) and Slattery and Rayner (2010) undertook comparative analyses of reading across varied font types, and their findings highlighted the existence of a reading cost associated with unfamiliar fonts, evidenced by more and longer word fixations, especially when juxtaposed with familiar fonts. Additionally, recent studies showed that while font legibility influences reading metrics, readers can adaptively modify their eye movement patterns to the most effective reading strategy for the used font (Minakata and Beier, 2021). Moreover, reductions of text contrast in early reading stages have been shown to considerably increase fixation durations (Drieghe, 2008). Similarly, significant contrast reductions impair reading performance (Jainta et al., 2017). It is worth noting that, while optimal reading performance is ideally obtained in quiet, distraction-free environments, such conditions are rare in daily life. External auditory stimuli, be it the hum of traffic, ambient music, or overheard conversations, can intrude upon the reading experience, causing reading disruptions and potentially hampering comprehension (Vasilev et al., 2019). Likewise, within digital environments, irrelevant visual stimuli such as pictures or pop-up advertising can be detrimental, resulting in reading and comprehension costs (Copeland and Gedeon, 2015).

Visual degradation of the text and noisy print allow researchers to probe the resilience and adaptability of reading processes under suboptimal conditions, mimicking real-world scenarios. Jordan et al. (2003) employed a subtle manipulation by visually degrading letters, revealing that the degradation of exterior letters—i.e., letters situated at the outer edges of the word—led to a significant reduction in reading speed. Similarly, Gagl et al. (2014) further explored word processing by visually degrading parafoveal previews and showed a marked reduction in reading speed. Thus, degraded previews, especially of critical parts of words, can disrupt the fluidity of reading, implying longer fixations and potentially affecting comprehension (see also Johnson et al., 2007).

Due to the complexity of human perception, the task of isolating variables in cognitive research has always been a concern in the pursuit of creating valid and controlled experiments. The applicability and generalizability of findings derived from the constraining conditions of a laboratory setting have been a topic of debate, often termed the ‘real-world or the lab dilemma’ (Holleman et al., 2020). In this sense, VR comes as a viable tool for cognitive research to overcome some of the limitations of in-lab studies, as it seeks to simulate world events in a realist and immersive manner (Abdillah et al., 2020), mimicking more natural perceptual processes (Scarfe and Glennerster, 2019). Its immersive characteristics mitigate external distractors and are optimally suited for testing reading behavior, including eye movement patterns (Adhanom et al., 2023), by allowing the investigation of the interaction of reading stimuli with environmental attributes, crucial to determine the key factors influencing human cognition in-the-wild and to establish benchmarks for designing outdoor reading material.

The use of VR for psychological experiments is still at a relatively incipient stage. Yet, researchers have reliably replicated patterns of classic cognitive effects in VR, such as the effect of incongruent flankers (Jubran et al., 2022; Rocabado and Duñabeitia, 2022), lexicality effects (Mirault et al., 2021) and other reading indices (Mirault et al., 2020). Research comparing VR and PC methods of stimuli presentation have shown no significant difference between the two (Jubran et al., 2022), suggesting that VR is a viable method for conducting cognitive research as long as certain critical aspects are taken into account, like the visual angles used for stimuli presentation and the methods used for response collection. Likewise, VR reading experiments should consider other aspects such as the resolution, in terms of pixel density, refresh-rate and field-of-view, or other parameters of the text (e.g., size, font, location), given that these are factors that can alter reading experience and are improving continuously with technological advances (e.g., Dingler et al., 2018; Dittrich et al., 2013; Lee et al., 2022; Rzayev et al., 2021). Nevertheless, as technological advancements continue to reshape the research landscape, innovations like VR stand out, offering opportunities to investigate reading under crafted experimental conditions (Mirault et al., 2020).

In the present study, we investigated text-background contrast in reading comprehension. Drawing on historical studies (Tinker and Paterson, 1931; Timmers, 1978), we highlight the evolving importance of this factor in the context of modern technologies like computer screens (Mills and Weldon, 1987) and augmented reality displays (Gabbard et al., 2006). The effects of contrast on reading have been a topic of research for many years (e.g., Legge et al., 1987). Although contrast needs to be substantially reduced to affect reading performance, there is consensus that diminished contrast levels can increase the time needed for text processing (Legge et al., 1987; Drieghe, 2008; Brychtova and Coltekin, 2016; Jainta et al., 2017). By comparing this element with a manipulation of environmental noise, our study explores the dynamics of word recognition in real-world scenarios, where stimuli can be degraded under unpredictable weather conditions. Thus, the present study included real-world elements and examined the interaction of visual disturbances stemming from diverse sources: those intrinsic to the linguistic input, such as two contrast levels (high and low), and those stemming from the environment, specifically weather conditions (rainy and sunny). By employing both a Lexical Decision Task and a Sentence Reading Tas within a 3D environment, we aimed to broaden our understanding of the reading dynamics under diverse conditions, capturing effects on both single-word and sentence processing in comparable experimental conditions. Considering previous findings, we expected manipulations related to low contrast, particularly under adverse weather conditions, to reduce processing efficiency and increase error rates. Moreover, central to this study are the exploration of how word and sentence reading processes are influenced by both script contrast-related noise and external environmental disturbances, and the investigation of whether real-world visual challenges, such as reading through a rain-smeared window, capture and replicate the effects typically seen in laboratory settings related to reading disfluency in contrast manipulations.

## 2 Experiment 1: Single-word recognition

The first experiment used the Lexical Decision Task to explore single-word recognition in different contrast and weather conditions in VR. The main effects of contrast (high vs. low) and weather conditions (sunny vs. rainy), as well as their potential interaction, were analyzed. We predicted that low contrast, particularly in adverse weather conditions, would reduce processing efficiency resulting in longer decision times and increased error rates.

### 2.1 Methods

#### 2.1.1 Participants

A total of 40 university students and employees from Nebrija University, who were native Spanish speakers, participated in this study for monetary compensation. They all had normal or corrected-to-normal visual acuity and hearing. None showed cognitive impairments in the Cognitive Assessment Battery (CAB) PRO (CogniFit Inc., San Francisco, CA). 26 of the participants self-identified as female (Mean age = 24.31, SD = 9.99) and 14 participants self-identified as male (Mean age = 24.5, SD = 4.35). *A priori* power analyses were conducted using G*Power version 3.1.9.7 (Faul et al., 2009). Given the exploratory nature of the present study and the aim to detect meaningful differences across conditions, a medium effect size was considered appropriate. Therefore, for a medium effect size (*f* = 0.25), to achieve a power of 0.95, at a significance criterion of *α* = 0.05, the minimum sample size estimated was *N* = 36 for a repeated-measures ANOVA analysis. Participants were granted written informed consent for their participation in accordance with the Declaration of Helsinki. The experimental procedures were approved by the Research Ethics Committee at Nebrija University (approval code UNNE-2022-0017).

#### 2.1.2 Stimuli

For the construction of the Lexical Decision Task, a total of 400 six-letter items were selected, including both words and pseudowords. These items were sourced from the SPALEX database, a repository stemming from a Spanish crowdsourced lexical decision mega-study (Aguasvivas et al., 2018). All selected words were high-frequency nouns. Their mean Zipf frequency, derived from the EsPal corpus (Duchon et al., 2013), was 4.52, ranging between 3.48 and 5.91. Selected words had a recognition accuracy of 100% in the SPALEX corpus. The pseudowords were also obtained from SPALEX, and while maintaining a recognition accuracy of 100%, as expected considering lexicality effects, they had a longer mean RT compared to word stimuli in the database: 1073 vs. 783 ms.

The visual presentation of all stimuli was set against a white background (RGB: 255, 255, 255). Stimuli were rendered in lowercase using the Courier monospaced typeface. For the high contrast condition, the lettering was in stark black (RGB: 0, 0, 0), while for the low contrast, a muted shade (RGB: 156, 156, 156) was employed. This specific shade has been identified to impede reading speed (Yu et al., 2022). On average all stimuli subtended horizontally 10.49° of visual angles from the participants’ viewpoint.

**Figure 1:**
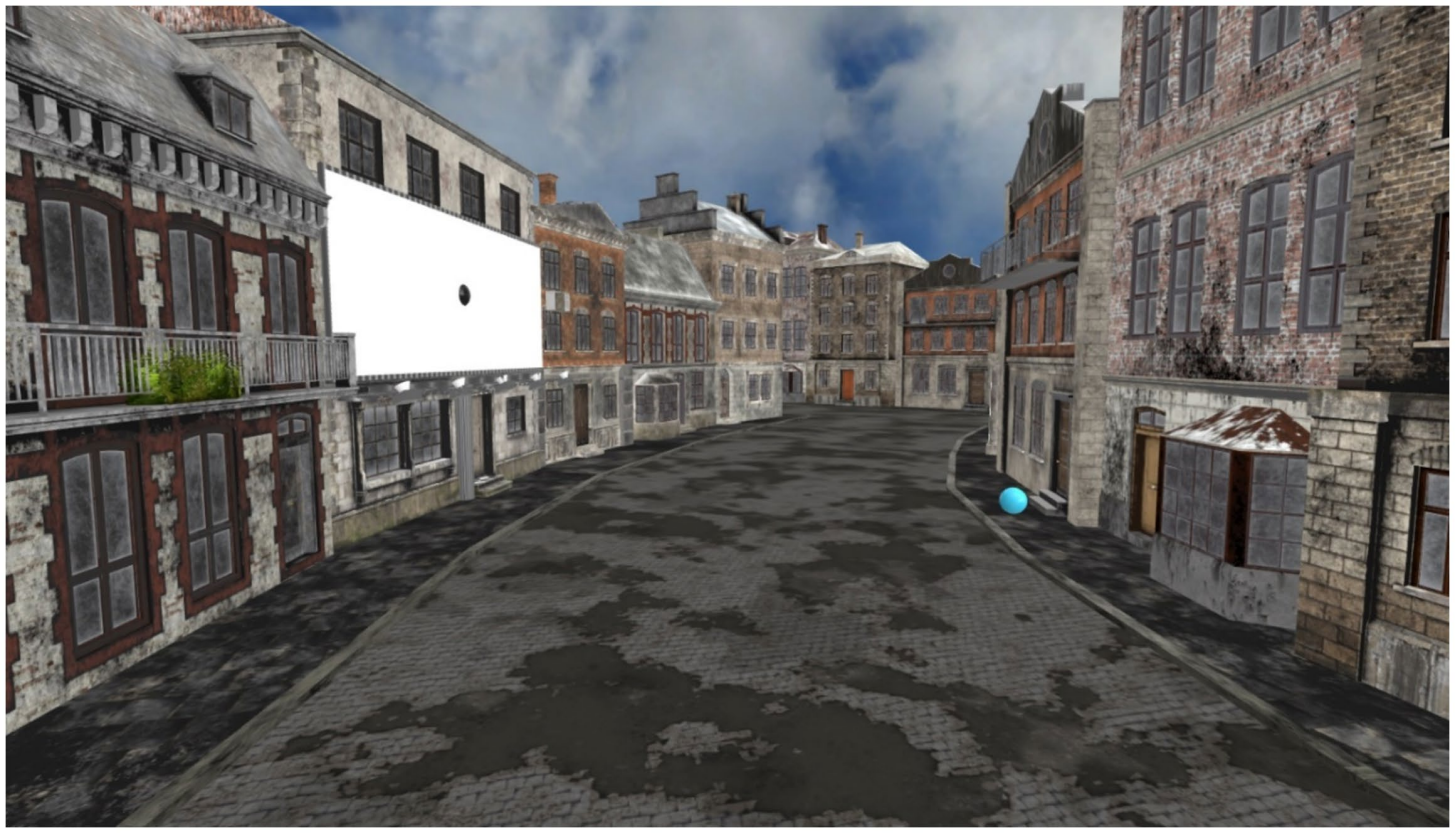
Overview of the experimental setup: distribution of 3D models and virtual environment layout. Participants’ location is indicated with a blue dot.

**Figure 2:**
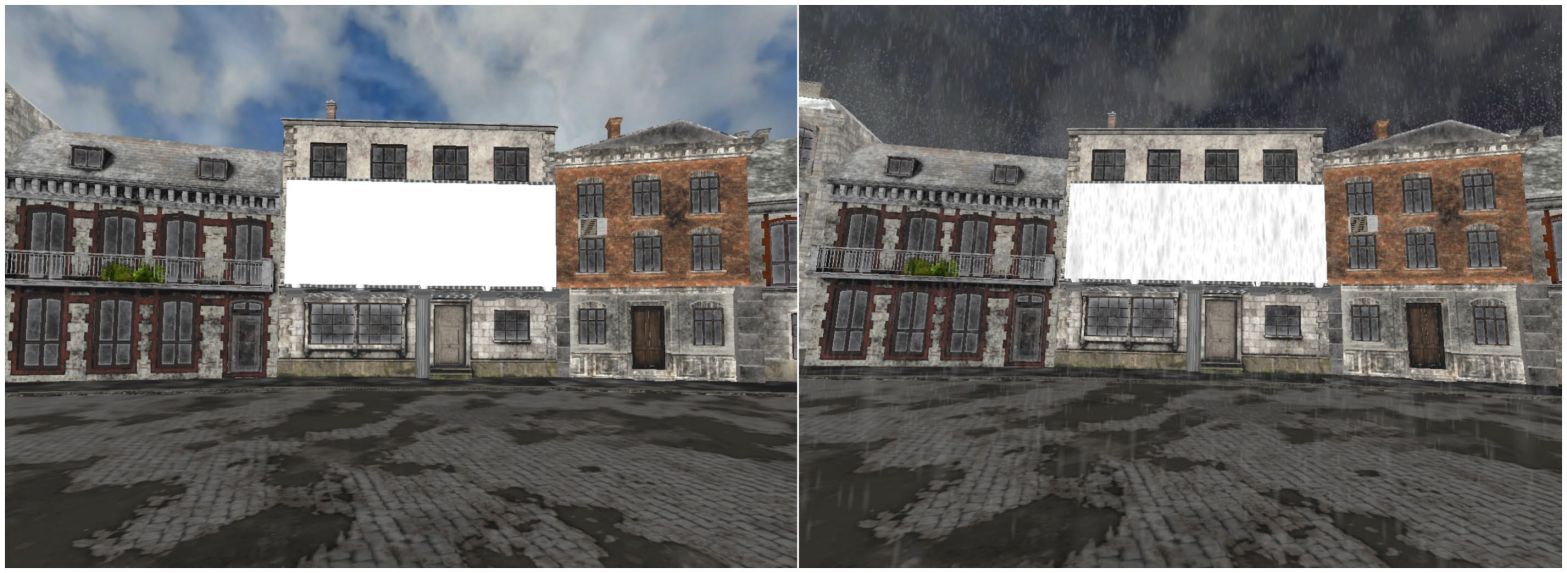
Participant’s visual perspective in Experiments 1 and 2. An example of the sunny weather condition is displayed on the left and an example of the rainy weather condition is shown on the right.

The virtual environment was created using open-access 3D models imported from Sketchfab. Background animations, such as sky movement or idle movement for characters (e.g., the piggles) were added to enhance immersion. Redundant 3D elements were removed from the main models using Blender, and all stimuli were displayed on a 3D billboard integrated into the virtual landscape. Stimuli were presented under two weather conditions: sunny and rainy. Raindrop animations were inserted for the rainy weather condition. For visual representations of these scenarios see Figures 1, 2 (see also supplementary material at https://osf.io/zcdnh/?view_only=d65e54d2ea774a8cbe7ccb30fda13794 for video demonstrations of the different tasks).

#### 2.1.3 Apparatus

The experiment was conducted on a high-performance gaming laptop computer, featuring an Intel Core i7-10750H processor (2.6 GHz), a Windows 10 operating system (64-bit), 32 GB of RAM, and an NVIDIA GeForce RTX 2070 graphics card to ensure a high-quality presentation. The Vizard 6 programming platform was used, which operates on a Python 2.7-based system (Worldviz, 2019). The VR experience was rendered by a HTC VIVE Pro HMD, with a resolution of 2880×1600 pixels and a field of view of 110°. To ensure optimal device performance and communication between the computer and the HMD, the battery-saving settings were disabled throughout the experiment, as well as the SteamVR Motion Smoothing system to maintain the refresh rate constant.

#### 2.1.4 Task and procedure

Participants were equipped with an HMD and immersed in a virtual three-dimensional environment, seated on a rotating chair. This setup provided them with a stationary vantage point within the VR environment, facilitating a complete 360-degree rotational view. To ensure optimal visual clarity and comfort, participants were guided to adjust the HMD’s positioning and the eye-to-eye alignment. This procedure was followed by a 5-point gaze fixation calibration, integrated in eye tracker of the HMD.

**Figure 3:**
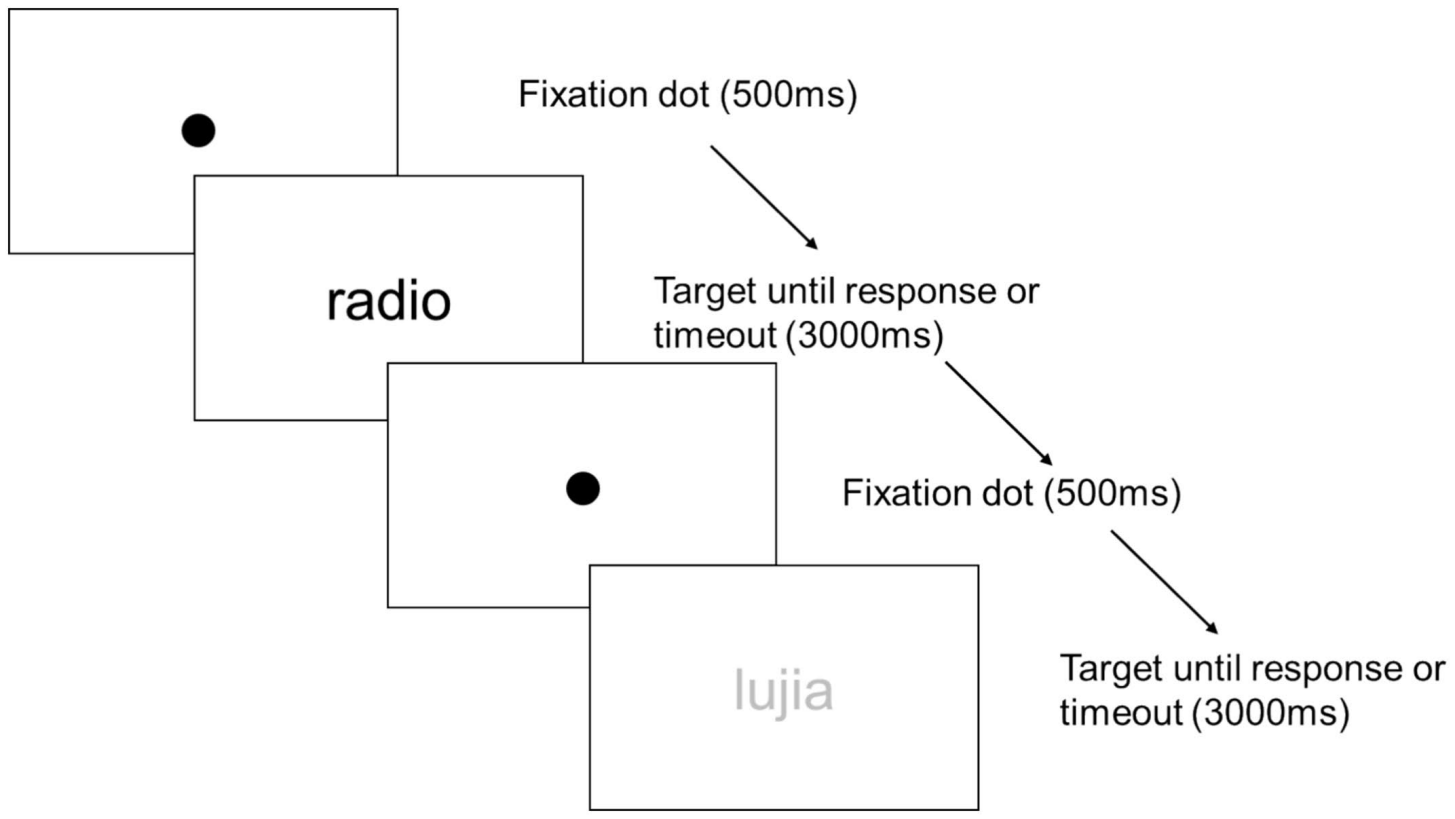
Representation of the structure of two sequential trials in Experiment 1 in high- and low-contrast conditions, respectively.

**Figure 4:**
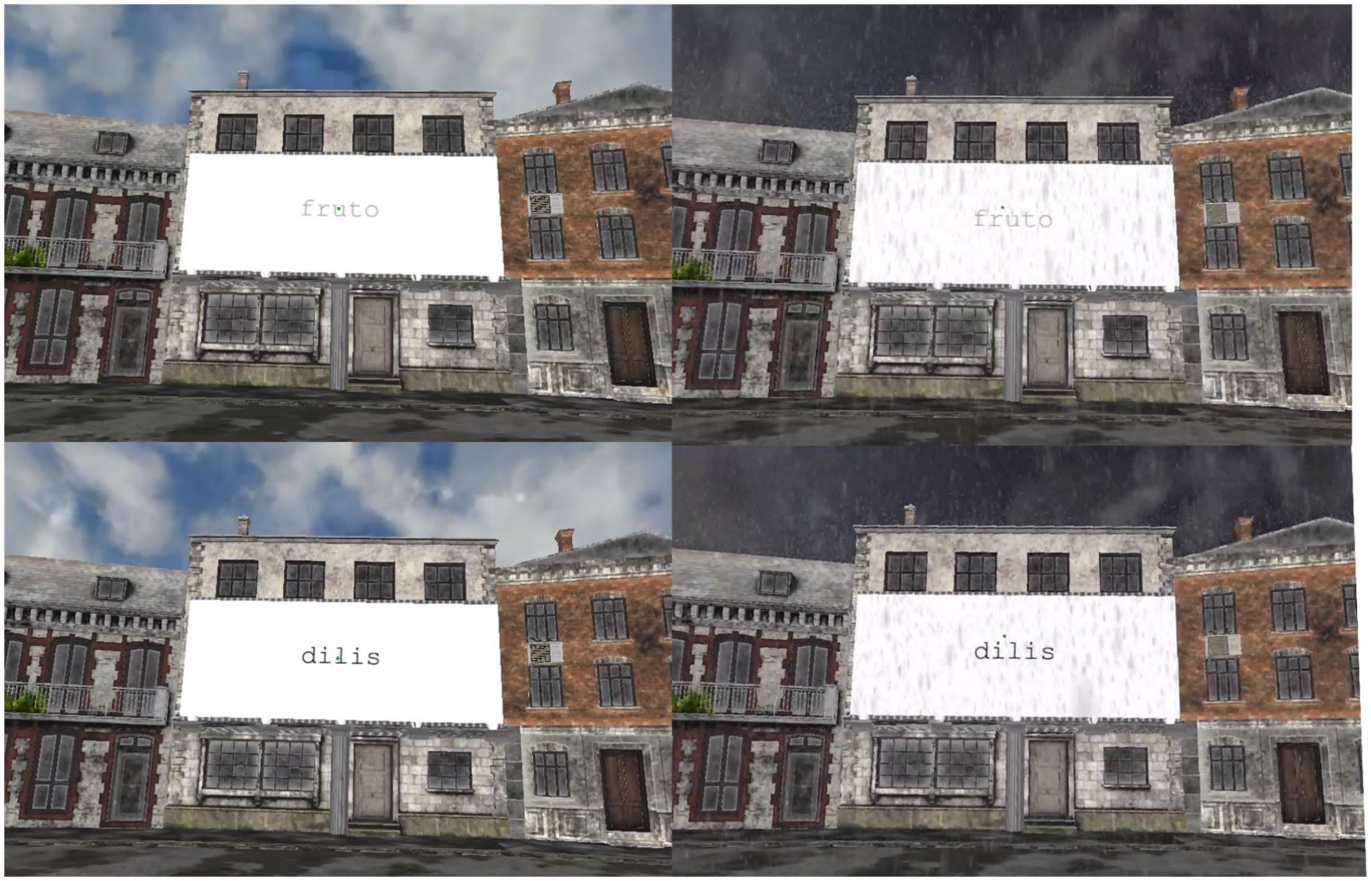
Participant’s visual perspective during Experiment 1. Examples of a low contrast word and high contrast pseudoword under sunny weather conditions are displayed on the left and examples of a low contrast word and high contrast pseudoword under rainy weather conditions are shown on the right.

Participants were instructed to determine whether the presented letter strings were legitimate Spanish words or not. They responded using the VR controllers, pressing the right trigger for words and the left trigger for pseudowords. The task included 200 items per weather condition (100 words, 100 pseudowords). Reaction times and accuracy were recorded. Each trial began with a 500-millisecond fixation point, followed by the word or pseudoword, which remained on display until a response was made or for a maximum of 3,000 milliseconds. For a visual representation of the trial structure see Figure 3. The stimuli in each block (rainy, sunny) were presented in a random order for each participant, and the presentation order of the blocks were also randomized across participants. Two lists were created so that each item could appear only in one condition for each participant, but the conditions were counterbalanced across participants to avoid any list-specific effect. For a visual exemplar of the task see Figure 4.

#### 2.1.5 Data processing and analysis

Four participants were excluded from the final analysis due to errors in data collection. The final sample was thus *N* = 36 with 25 participants who self-identified as female (Mean age = 24.48, SD = 10.17) and 11 who self-identified as male (Mean age = 23.92, SD = 4.10).

The data was preprocessed using R (R Core Team, 2022) in RStudio (RStudio Team, 2022). RTs below 300 ms and those that were 2.5 standard deviations faster or slower than the mean RT per condition and per participant were excluded. This process resulted in a rejection of 3.16% of data belonging to the rainy condition and 2.94% from the sunny condition. Accuracy was defined as the percentage (%) of correct responses per participant throughout the task in each condition. Exploratory analyses showed that estimated marginal mean probabilities on accuracy rates for words were almost at ceiling and highly similar across conditions (see Table 1). For this reason, only data RT were further analyzed.

**Table 1:** Mean accuracy rates and response times in milliseconds for words and pseudowords across experimental conditions.

		Words		Pseudowords	
Contrast condition	Weather condition	Reaction times M (SD)	Accuracy M (SD)	Reaction times M (SD)	Accuracy M (SD)
High contrast	Sunny weather	618 (167)	0.97 (0.18)	717 (218)	0.97 (0.18)
Low contrast	Sunny weather	630 (177)	0.97 (0.17)	728 (226)	0.98 (0.14)
High contrast	Rainy weather	631 (168)	0.96 (0.20)	726 (237)	0.96 (0.19)
Low contrast	Rainy weather	646 (168)	0.97 (0.17)	730 (238)	0.98 (0.15)

### 2.2 Results

A linear mixed-effects model was used to analyze the data related to word stimuli. The model had RT (in milliseconds) as a dependent variable (*N* = 6,612 observations) and included a fixed-effects structure consisting of the two-level factors Contrast (high vs. low) and Weather (sunny vs. rainy), as well as their interaction. To account for variability across participants and items, the random-effects structure included random intercepts for both Participants and Items. This structure was selected as the simplest model capable of explaining the data while controlling for individual differences and item variability without overfitting. Other more complex random structures, including additional random slopes, were also tested; however, these models did not converge, supporting the selection of the final random structure. The model’s random structure included random intercepts for Participants and Items. The model formula (in R notation) was as follows: Reaction Time ~ Contrast * Weather + (1 | Subject) + (1 | Item). See Table 1 for descriptive analysis results.

**Figure 5:**
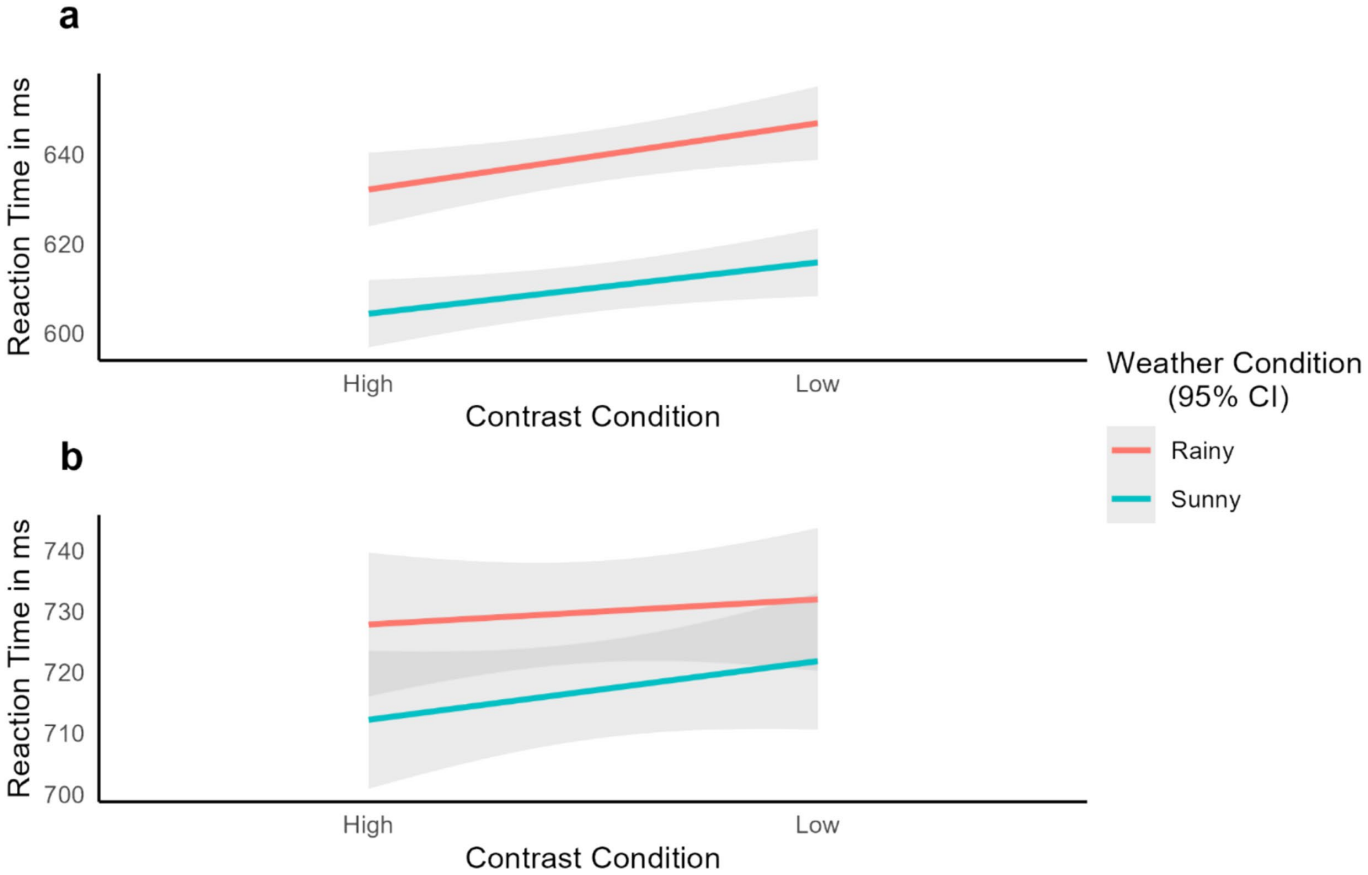
Experiment 1 reaction time results in the different experimental conditions for (a) words and (b) pseudowords. Smooth lines represent the fit from a linear mixed-effects model estimating the effects of each factor and shaded areas represent 95% confidence intervals.

The model was run in Jamovi (The Jamovi Project, 2022) using the GAMLj module (Gallucci, 2019), and model convergence was achieved, confirming the appropriateness of the model for the data. The main effect of Contrast was found to be significant *F*(1, 186) = 4.88, *p* = 0.028, with RTs being shorter for high-contrast words than low-contrast words. Similarly, the effect of Weather was significant *F*(1, 6,389) = 28.44, *p* < 0.001, with sunny conditions yielding shorter response latencies as compared to rainy conditions. Finally, the interaction between the two manipulations was not significant, F(1, 6,389) = 0.52, *p* = 0.471 (see Figure 5A).

A similar linear mixed-effects model was constructed to analyze the pseudoword data. This included RT (in ms) as the dependent variable (n = 6,673 observations), Contrast (high vs. low) and Weather (sunny vs. rainy), and their interaction as fixed factors, and random intercepts for Participants and Items: Reaction Time ~ Contrast * Weather + (1 | Subject) + (1 | Item; see Table 1 for the descriptives).

Neither of the fixed factors nor their interaction were significant, with only Weather approaching significance: Contrast *F*(1, 197) = 0.61, *p* = 0.437; Weather *F*(1, 6,467) = 3.82, *p* = 0.051; Interaction F(1, 6,467) = 0.59, *p* = 0.441. The close-to-significance Weather effect showed that pseudowords in rainy environments were responded to slower than those in sunny conditions (for visualization, see Figure 5B).

### 2.3 Discussion

These results showed that visual text-background contrast and weather conditions significantly influence word recognition speed, while having little impact on pseudoword recognition. The significant main effect of contrast on word recognition speed, with high-contrast words showing faster RTs than low-contrast words, aligns with the notion that high visual contrast facilitates cognitive processing due to enhanced visual clarity (Legge et al., 1987). This idea is further supported by the finding that sunny weather conditions, which presumably offer better lighting and hence better visual clarity, also yield faster latencies.

Results for the pseudoword data display a different pattern. The absence of a significant effect of contrast manipulation on pseudoword recognition speed suggests that the facilitative effect of high visual contrast may be specific to recognizable, meaningful stimuli like words. This distinction between words and pseudowords may be rooted in cognitive processing differences between meaningful and non-meaningful stimuli, as highlighted by Oppenheimer (2008). Nonetheless, the close-to-significant effect of weather conditions on pseudoword processing hints at a broader influence of environmental factors on cognitive processing.

Moreover, the lack of interaction between contrast and weather conditions in both word and pseudoword recognition suggests that these factors operate independently in influencing recognition latencies. This independence implies that the effects of visual contrast and environmental conditions on reading are not contingent on each other, and provides insight on how different features and environmental factors affect cognitive processing differently.

## 3 Experiment 2: Sentence reading

Experiment 2 was created to examine sentence reading for comprehension and eye-gaze fixation, assessing how script-associated physical noise and external environmental disturbances impact reading in a setting like that used in Experiment 1. We hypothesized that rainy weather and lower contrast would increase fixation durations and decrease sentence comprehension.

### 3.1 Methods

#### 3.1.1 Participants

The same participants as in Experiment 1 completed Experiment 2. (Note that Experiments 1 and 2 were conducted in the same session).

#### 3.1.2 Stimuli

Two hundred simple transitive sentences (subject + verb + predicate; e.g., The chicken crossed the road), with an average of 37.39 characters in length, were used as stimuli. Each character subtended 0.67 visual angle degrees. The latest version of ChatGPT (GPT-4, OpenAI, 2023) was used to assist with sentence creation. Once these were generated, each of them was human-assessed to ensure its validity. Furthermore, 40 yes/no comprehension questions were randomly created for 40 of the sentences.

**Figure 6:**
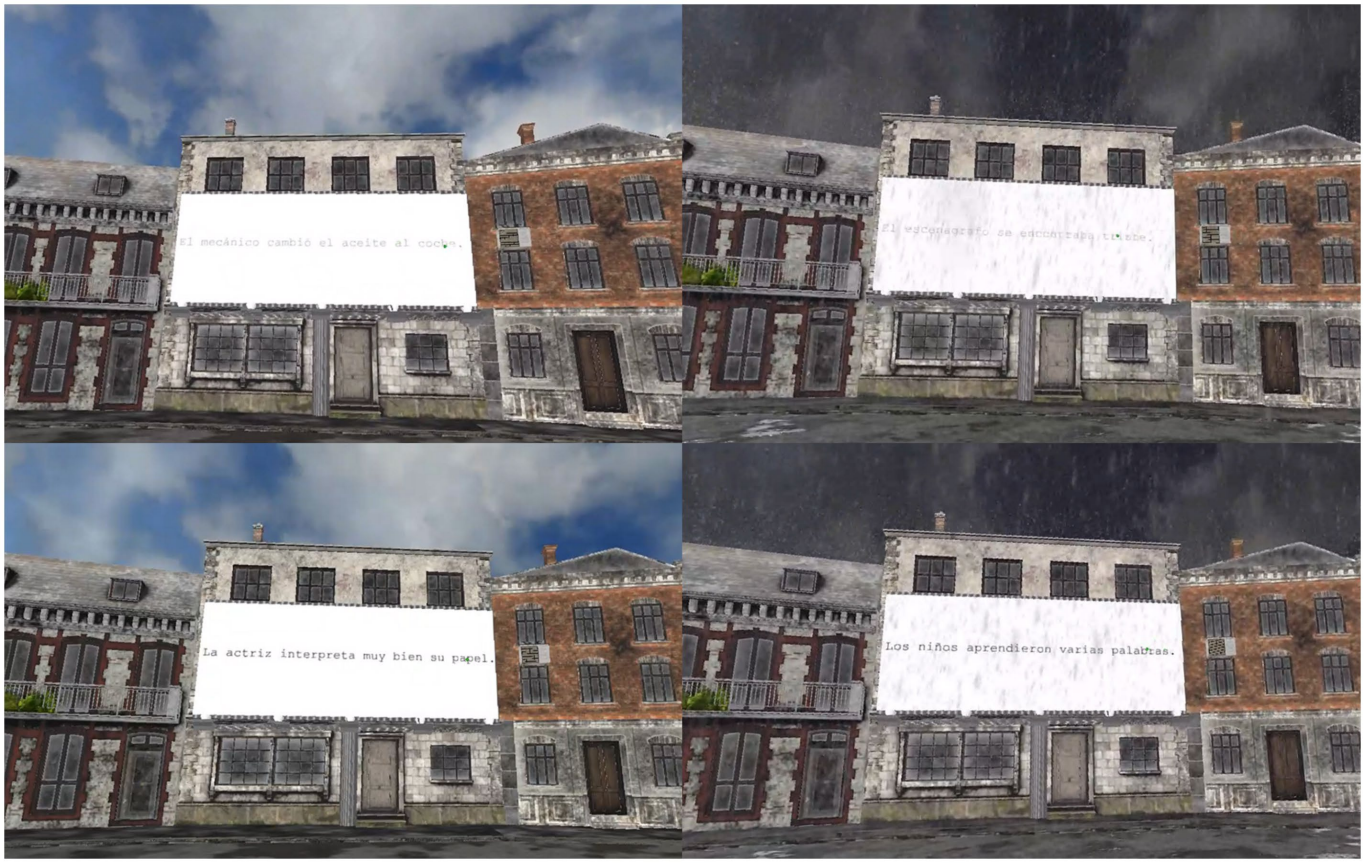
Participant’s visual perspective during Experiment 2. Sentence examples in high and low contrast under sunny weather conditions are displayed on the left and sentence examples in high and low contrast under rainy weather conditions are shown on the right.

Each sentence was presented in either a high or low contrast condition, following the same presentation procedure as in Experiment 1. Two lists were created with 50 sentences in each of the conditions (i.e., high contrast and rainy environment, low contrast and rainy environment, high contrast and sunny environment, low contrast and sunny environment). The sentences were counterbalanced across conditions and participants to control for any potential effects associated with specific stimulus properties. Comprehension questions were split evenly between high and low-contrast sentences and were presented in black text. All stimuli were presented within the same 3D environment model as in Experiment 1, under the same two weather conditions: sunny and rainy (see Figure 6).

#### 3.1.3 Apparatus

The experiment was run through the same apparatus as Experiment 1, under the same settings.

#### 3.1.4 Task and procedure

**Figure 7:**
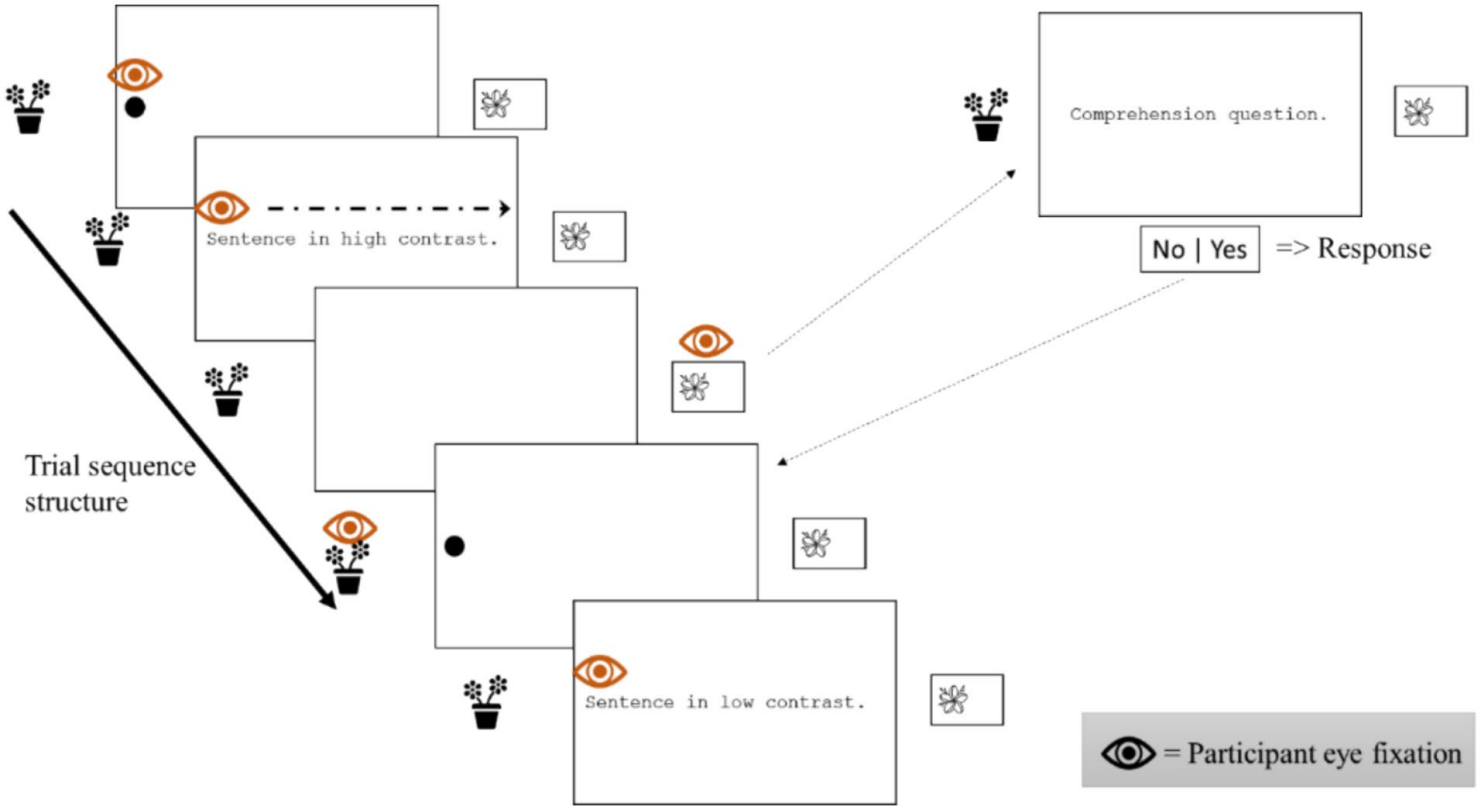
Representation of trials and gaze interactions in Experiment 2.

Sentences were presented through the same HMD. Before starting the experiment, the built-in eye tracking system was submitted to a calibration accuracy assessment, with subsequent re-calibration processes if it was needed. Instructions were presented in a text box, directing participants to read each sentence at their own pace, avoiding overreading yet paying attention as comprehension questions were going to be presented along the task. The task was designed to display every sentence after participants had fixated their gaze on a fixation dot placed at the beginning of each sentence. Once a sentence was read, participants had to gaze-interact with different objects of interest presented along the main scenario: (1) an air conditioning compressor hanging under a window to mark the end of reading and (2) ornamental plants placed on a balcony at the left side of the billboard to end the trial and initiate the next one. Before starting the experiment, participants were allowed to get familiar with the scenario and its different components. Twenty of the comprehension questions required the participant to say “yes” by pulling the right trigger. For the other 20, the correct response was “no,” indicated by pulling the left trigger. Ten of each of the comprehension question types were related to sentences on each contrast condition. Figure 7 schematically represents gaze and task interactive procedures. As in Experiment 1, stimuli were presented in a random order. Stimuli lists were counterbalanced across weather conditions, and weather conditions were counterbalanced across participants.

#### 3.1.5 Data processing and analysis

Reading data was preprocessed and cleaned using R (R Core Team, 2022) with the emov package (Schwab, 2016) within RStudio (RStudio Team, 2022) for measuring fixation durations and locations. This package implements a dispersion-based algorithm (I-DT), rather than relying solely on the velocity of eye movements as some conventional algorithms do. This emphasizes the spatial dispersion of consecutive data points over time (Salvucci and Goldberg, 2000). Specifically, if the dispersion of these points remains below a predetermined threshold for a certain duration, such an event is classified as a fixation. This approach allows for the assessment of both the durations and spatial positions of fixations in eye-tracking data.

### 3.2 Results

Separate analyses were conducted for each eye-tracking measure: total reading time, fixation duration, and number of fixations. This approach allows for a robust analysis of the effects of contrast and weather conditions on different eye movement measures. Linear mixed-effects models (LMMs) were used to analyze the data, with the goal of accounting for variability both across participants and items. The random-effects structure included random intercepts for Participants and Items, which allowed for individual differences in reading behavior and variations in item difficulty. This random structure was selected to ensure that the model could generalize across both participants and items. Similar to experiment 1, complex random structures including random slopes were tested. However, these models failed to converge, leading to the selection of a simpler random-effects structure leading to the selection a simpler random-effects structure that still captured the essential variability without overfitting. Thus, the fixed-effects structure included the two-level factors Contrast (high vs. low) and Weather (Sunny vs. Rainy), as well as their interaction, to assess their impact on the dependent variables. The model formulas, in R notation, were as follows: Dependent Variable ~ Contrast * Weather + (1 | Subject) + (1 | Item). All models were fit using the GAMLj module in Jamovi (The Jamovi Project, 2022; Gallucci, 2019). Model convergence was assessed, and all models converged successfully, ensuring that the parameter estimates were reliable. See Table 2 for an overview of the descriptive analysis results.

**Table 2:** Descriptive statistics for total reading time in milliseconds (ms), fixation duration in milliseconds, and number of fixations across weather and contrast conditions.

		Total reading time	Fixation duration	Number of fixations
Contrast condition	Weather condition	M (SD)	M (SD)	M (SD)
High contrast	Sunny weather	1898 (969)	161 (23.3)	6.57 (2.77)
Low contrast	Sunny weather	1913 (959)	161 (23.3)	6.62 (2.91)
High contrast	Rainy weather	1994 (994)	162 (24.4)	6.77 (2.80)
Low contrast	Rainy weather	2062 (1015)	161 (24.5)	6.83 (2.85)

#### 3.2.1 Comprehension questions

Overall comprehension accuracy was 96.35% (Mean accuracies across conditions between 95.8 to 96.8%). No differences were observed across conditions (Fs < 1).

#### 3.2.2 Total reading time

**Figure 8:**
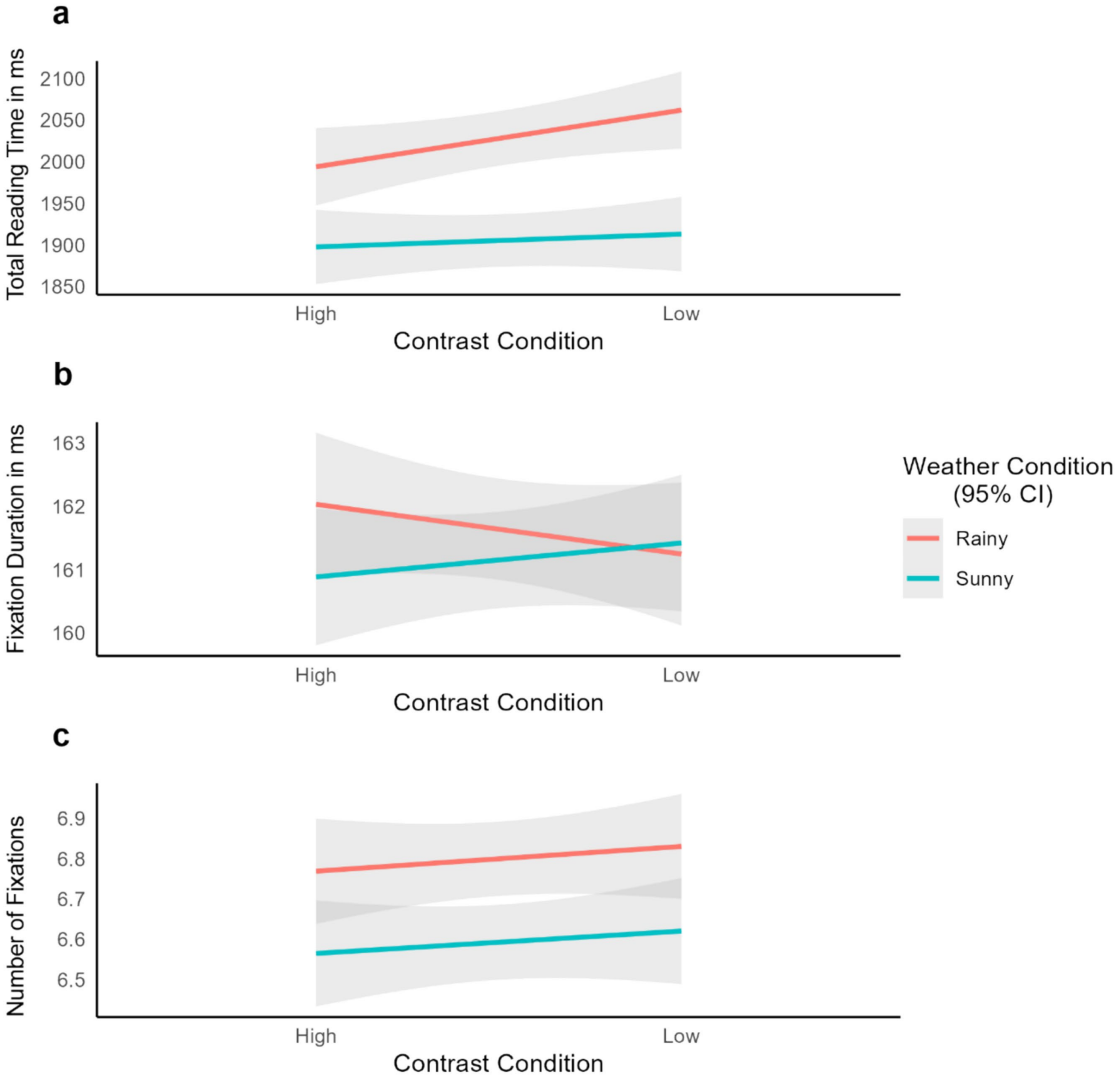
Experiment 2 results across Contrast and Weather conditions for (a) Total Reading time, (b) Fixation Duration, and (c) Number of Fixations. Smooth lines represent the fit from a linear mixed-effects model and shaded areas represent 95% confidence intervals.

The analysis revealed a non-significant main effect of Contrast, *F*(1, 199) = 1.41, *p* = 0.236. However, a significant main effect of Weather was observed, *F*(1, 7,030) = 35.30, *p* < 0.001, with a mean difference of 123 ms indicating shorter reading times under sunny conditions as compared to rainy conditions. The interaction between Contrast and Weather conditions was not significant, *F*(1, 7,028) = 2.26, *p* = 0.133 (see Figure 8A).

#### 3.2.3 Fixation duration

The main effect of Contrast was not significant, *F*(1, 197) = 0.101, *p* = 0.751. Similarly, the main effect of Weather conditions was not significant, *F*(1, 7,113) = 0.930, *p* = 0.335. The interaction between contrast and weather conditions was also not significant, F(1, 7,113) = 1.803, *p* = 0.179 (see Figure 8B).

#### 3.2.4 Number of fixations

The analysis showed a non-significant main effect of Contrast, *F*(1, 198) = 0.44, *p* = 0.507. However, a significant main effect of Weather was observed, *F*(1, 7,044) = 13.40, *p* < 0.001, with a mean difference of 0.22, indicating a higher number of fixations under rainy conditions as compared to sunny conditions. The interaction between Contrast and Weather was not significant, *F*(1, 7,042) = 0.05, *p* = 0.826 (see Figure 8C).

### 3.3 Discussion

Experiment 2 investigated sentence reading comprehension and dynamics under varying contrast and weather conditions, aiming to understand how these factors influence reading behavior. Starting with comprehension questions, participants exhibited a very high accuracy rate. Notably, neither contrast nor weather conditions significantly influenced comprehension accuracy. This suggests that, while participants might have experienced variations in reading times under different conditions, their ability to comprehend the content remained largely unaffected, in line with previous evidence (Vasilev et al., 2019).

In terms of total reading times, our findings revealed a significant main effect for weather conditions, with sunny weather decreasing reading times compared to rainy weather. This aligns with the intuitive understanding that environmental distractions, such as simulated rain, can emulate results from previous research in which blurred sentences showed a reading cost (e.g., longer reading times; Chung et al., 2007).

Lastly, the fact that the fixation duration analysis revealed no significant effects for either contrast or weather conditions, and that contrast did not affect the number of fixations either, suggests a different effect pattern compared with previous research that manipulated text font clarity (Slattery and Rayner, 2010). Notwithstanding, the number of fixations was significantly influenced by weather conditions, with rainy weather leading to a higher number of fixations. This indicates that environmental factors might subtly influence these precise eye movement patterns during reading.

In conclusion, findings from Experiment 2 emphasize the multifaceted influence of environmental visuoperceptual factors on reading dynamics in a VR setting. While Experiment 1 emphasized the relevant role of contrast and weather conditions in reading efficiency, results from Experiment 2 suggest that environmental factors like simulated weather conditions might play a more pronounced role in influencing sentence reading performance in VR.

## 4 General discussion

The two experiments reported here examined the interplay between visual contrast, environmental conditions, and their collective impact on word recognition and reading dynamics. The findings align with the body of literature that emphasizes the role of visual and environmental factors in modulating cognitive and reading processes.

In the first experiment, the significant main effect of visual contrast found on word recognition speed resonates with the established understanding that high visual contrast facilitates cognitive processing by enhancing visual clarity (Legge et al., 1987). The favorable impact of sunny weather conditions on word recognition further accentuates the potential of environmental factors in modulating cognitive processing times. However, the differential impact observed for pseudoword recognition suggests a nuanced mechanism, possibly rooted in the cognitive processing disparities between meaningful and non-meaningful stimuli as suggested by Oppenheimer (2008). The lack of interaction between visual contrast and weather conditions indicates that these effects that have been found to impact reading times potentially operate independently from each other in influencing single-word recognition.

The second experiment investigated sentence reading comprehension and reading dynamics, showing a significant main effect of weather conditions on total reading times. Despite variations in reading times under different conditions, the almost-at-ceiling comprehension accuracy underscores the resilience of comprehension processes, aligning with previous findings (Vasilev et al., 2019). The divergence in effect patterns concerning fixation duration and number of fixations compared to previous research (Slattery and Rayner, 2010) hints at the complex interaction of visual and environmental factors in reading dynamics, at least in a VR setting.

While this study focused on creating ecologically valid virtual environments that can emulate real-life situations and conditions, there are technical limitations associated with VR that should be acknowledged as they could have potentially influenced participants’ reading behavior. In this study, efforts were made to simulate realistic environmental conditions. Accordingly, the rainy scene was adjusted for drop velocity and light conditions, in a texture that covered the whole scenario, with drops that continuously scrolled following the direction of the falling rain. Thus, the raindrops crossed the target stimuli, as they would do in the real-world, and these visual elements were presented in the scene together with coherent auditory elements. This necessarily implied that lighting and sound conditions were nonidentical between the rainy and sunny environments, as it would happen in real-life scenarios. In order to account for these differences, the sunny environments also included certain sounds that are typically identified with sunny days, such as the sound of a fountain and a bird. The presence of lighting and sound variability between weather contexts represents an unavoidable difference that we deem necessary as part of the manipulations since these differences are inherent and necessary when designing experiments that aim to emulate real-life conditions, which are, by definition, multifactorial and intrinsically variable.

Some other limitations and technical issues are worth acknowledging. As noted in previous research, reading in VR can be affected by properties such as resolution, pixel density and field of view, which may differ from properties affecting traditional in-lab 2D reading environments (Scarfe and Glennerster, 2019). While these factors can hinder processing leading to slower reading speeds or increased visual strain, ongoing research has proposed solutions, such as adjusting text size, font, and viewing angles, to mitigate these effects (Dingler et al., 2018; Rzayev et al., 2021). These technical constraints, along with the complexity of simulating realistic visual and auditory scenes, must be considered when interpreting the findings and could stimulate further research.

By examining how reading performance is influenced in natural environments and evaluating the impact of contextual factors on legibility under different weather conditions, this study contributes to improving the design of outdoor reading materials, ensuring better visibility in real-world settings. Future research can expand this study by investigating the effects of rain on reading comprehension during dynamic tasks that involve motion (given that this is a usual condition in which we read while on the street, be it while walking, driving or on the bus), as well as the effects of different rain density (particularly relevant due to phenomena of crowding). Additionally, other phenomena like snow, fog, smoke, and haze should be studied, as they represent common meteorological conditions. Since the current experimental approach and method allows examining different populations, readers of varying levels of expertise could also be tested in further research. This is particularly noteworthy in the case of dyslexic population, as they have been found to be more affected by crowding and information density (see Bertoni et al., 2019 for a review). Additionally, the engaging, game-like ambiance of VR resonates especially with younger demographics, facilitating the exploration of reading dynamics in children (Mirault et al., 2021).

Taken together, these findings support the multifaceted nature of reading behavior, influenced not just by the intrinsic properties of the text, such as its physical characteristics like contrast, but also by the visuoperceptual properties of the surrounding environment caused by natural phenomena like meteorological conditions. The human cognitive system exhibits remarkable adaptability, from manipulations on single words (Perea et al., 2018) to sentences (Chung et al., 2007; Minakata and Beier, 2021). This study takes a step forward toward understanding the adaptability of the human reading system to naturalistic situations, increasing the conditions for the representativeness of effects initially observed in laboratory settings. While acknowledging the debates surrounding the artificiality and naturalness introduced by VR, studies like the current one highlight its potential as a powerful tool for investigating the interplay between specific environmental contexts and the diverse aspects of cognitive and complex behavioral functioning, such as language and reading.

## Data Availability

The datasets generated and analyzed during the current study are available in the Open Science Framework repository, accessible via the following link (anonymized): https://osf.io/zcdnh/?view_only=d65e54d2ea774a8cbe7ccb30fda13794.
